# Whole Organ Blood and Lymphatic Vessels Imaging (WOBLI)

**DOI:** 10.1038/s41598-018-19663-w

**Published:** 2018-01-23

**Authors:** Roni Oren, Liat Fellus-Alyagor, Yoseph Addadi, Filip Bochner, Hila Gutman, Shani Blumenreich, Hagit Dafni, Nava Dekel, Michal Neeman, Shlomi Lazar

**Affiliations:** 1 Department of Biological Regulation, Weizmann Institute of Science, Rehovot, 76100 Israel; 2 Department of Life Sciences Core Facilities, Weizmann Institute of Science, Rehovot, 76100 Israel; 3 Department of Pharmacology, Israel Institute for Biological Research, Ness Ziona, 74100 Israel; 4 Department of Biomolecular sciences, Weizmann Institute of Science, Rehovot, 76100 Israel; 5 Department of Veterinary resources, Weizmann Institute of Science, Rehovot, 76100 Israel

## Abstract

Thin section histology is limited in providing 3D structural information, particularly of the intricate morphology of the vasculature. Availability of high spatial resolution imaging for thick samples, would overcome the restriction dictated by low light penetration. Our study aimed at optimizing the procedure for efficient and affordable tissue clearing, along with an appropriate immunofluorescence labeling that will be applicable for high resolution imaging of blood and lymphatic vessels. The new procedure, termed whole organ blood and lymphatic vessels imaging (WOBLI), is based on two previously reported methods, CLARITY and Sca*l*eA2. We used this procedure for the analysis of isolated whole ovary, uterus, lung and liver. These organs were subjected to passive clearing, following fixation, immunolabeling and embedding in hydrogel. Cleared specimens were immersed in Sca*l*eA2 solution until transparency was achieved and imaged using light sheet microscopy. We demonstrate that WOBLI allows detailed analysis and generation of structural information of the lymphatic and blood vasculature from thick slices and more importantly, from whole organs. We conclude that WOBLI offers the advantages of morphology and fluorescence preservation with efficient clearing. Furthermore, WOBLI provides a robust, cost-effective method for generation of transparent specimens, allowing high resolution, 3D-imaging of blood and lymphatic vessels networks.

## Introduction

The study of complex anatomical structures, such as the blood and lymphatic vasculature, is dependent on improvements in imaging and computation techniques. An optimal imaging method for this purpose should provide the combination of high resolution and large 3D volume coverage. Classic histology, the gold standard analysis method, enables high spatial resolution over a large field. However, this method is limited to two-dimensional imaging of thin sections and may lead to misinterpretation upon attempting to conclude on a 3D structure. Optical imaging allows scanning of thicker tissues but is limited mostly by light scattering caused by various cellular and extra cellular structures. Available fluorescence-based scanning methods, such as confocal and two photon microscopies, provide high-resolution optical sectioning. Confocal microscopy can penetrate a few tens of micrometers into fixed fluorescently-labeled specimens and allows reconstruction of 3D images^1^. The use of two-photon microscopy further improves imaging penetration up to one millimeter^2^, but still covers relatively small fields of view. The more recent introduction of light-sheet technology made imaging of a few millimeters tissues possible, in a relatively short time and low energy^3^ making it favorable for advanced applications.

The recent advances in clearing techniques provide an additional step in optimizing tissue imaging, allowing deeper light penetration into specimens. The principle of transparent samples was first presented in 1914 by Spalteholz^4^, and a variety of tissue clearing methods were developed since^5^.

The currently available clearing methods can be roughly divided into two groups (as reviewed by Susaki *et al*.^6^): i) Organic solvents clearing methods, including 3DISCO^7–9^, iDISCO^10^, uDISCO^11^ and TDE^5,12^. These methods engage dehydration, delipidation and refractive index (RI) matching. ii) Hydrophilic clearing methods. While some of these methods such as SeeDB^13^ and Clear^T2 ^^14^, are based on RI matching by simple immersion, other methods including CUBIC^15^, Sca*l*eA2^16^, and Sca*l*eS^17^ involve hyper-hydration, delipidation or RI matching. Another subset of this group are gel hybridization-based clearing methods including CLARITY^18,19^ and PACT^20^ which have an additional step, in which a polymerized acrylamide (polyacrylamide) artificial skeleton in formed in the sample, prior to lipid clearance.

While performing tissue clearing and specifically in imaging of vasculature, the mode of fluorescent labeling should be addresed. Labeling can be achieved by either a reporter gene that expresses fluorescent protein or by immunolabeling (e.g. targeting PECAM, a marker of mature blood vessels^21^). Perfused blood vessels can also be labeled by intravenous injection of either targeted (e.g. lectin) or non-targeted (based on Dextran or other macromolecules) fluorescent contrast agents^21,22^. In contrast to the readily accessible blood vasculature, the difficulty in accessing lymphatic vessels makes the imaging of this system a challenging task (reviewed in^23^), requiring the use of targeted antibodies or reporter genes.

The advances in both, clearing techniques and microscopy, should allow imaging of large tissue specimens at high spatial resolution and provide details of intact networks, structures, and cell populations. Fine-tuning of these novel methods for imaging of the blood and lymphatic networks in whole organs, will greatly contribute to the generation of novel data regarding their role in physiological and pathological conditions as well as for the assessment of the response to vessel-targeted therapies^24^. Hence, the aim of this study was to develop a robust, affordable and scalable methodology for efficient tissue clearing, along with an appropriate immunofluorescence labeling that will be applicable for high resolution imaging of blood and lymphatic vessels networks. We termed this method, whole organ blood and lymphatic vessels imaging (WOBLI). The WOBLI procedure we propose here was built upon previously described CLARITY and Sca*l*eA2 techniques. We used the fluorescence marker tdTomato under the regulation of the Vecad promoter as a reporter of blood vessels, and immunolabeled LYVE1 as a marker for the lymphatic vessels. We report here that uterus, ovary, lung, liver and brain were efficaciously cleared using WOBLI while preserving both morphology and fluorescence intensity. We also successfully applied 3D light-sheet imaging of both lymphatic system and blood vasculature and were able to explore and quantify their structural features in the various organs using machine learning for vascular segmentation.

## Materials and Methods

### Materials

Neutral-buffered 4% paraformaldehyde solution (NB-PFA, pH = 7.0, Gadot, Israel) was used for tissue fixation. For hydrogel preparation, we used Photoinitiator VA-044 (Wako, USA), Acrylamide 40% solution and Bis- acrylamide 2% solution (Bio-Rad, Israel), Boric Acid, Urea, Glycerol, Triton X-100, Sodium hydroxide (NaOH) pellets and Sodium dodecyl sulfate (SDS) (Sigma, Israel). For immunolabeling the following antibodies were used: rabbit anti mouse LYVE1 antibody (Fitzgerald, USA); donkey anti rabbit Alexa fluor-647 antibody (Abcam, USA) and normal goat serum (NGS, Biological industries, Israel). To induce ovulation, pregnant mare serum gonadotropin (PMSG, National Hormone & Peptide Program, USA) was used.

### Animals

Experiments were carried out in accordance with the Israeli law and the Weizmann Institute guidelines. All experimental protocols were reviewed and approved by the Weizmann Institutional Animal Care and Use Committee.

Vecad^cre^ mice (B6.FVB-Tg(Cdh5-cre)7Mlia/J, Jackson) crossed with tdTomato^flox/stop/flox^ mice (B6.Cg-Gt(ROSA)26Sor^tm14(CAG-tdTomato)Hze/J^, Jackson) were used. In the double transgenic offspring, the red fluorescent protein variant (tdTomato) is controlled by vascular endothelial cadherin (VECAD) promoter, to be expressed only by endothelial cells of both developing and mature blood vessels. Ovaries and uteri were collected from 3-weeks-old mice, 24 hours after injection of pregnant mare serum gonadotropin PMSG, (5 IU). Lungs and livers were collected from 8-weeks-old mice.

### Whole Organ Blood and Lymphatic vessels Imaging (WOBLI)

**Figure 1:**
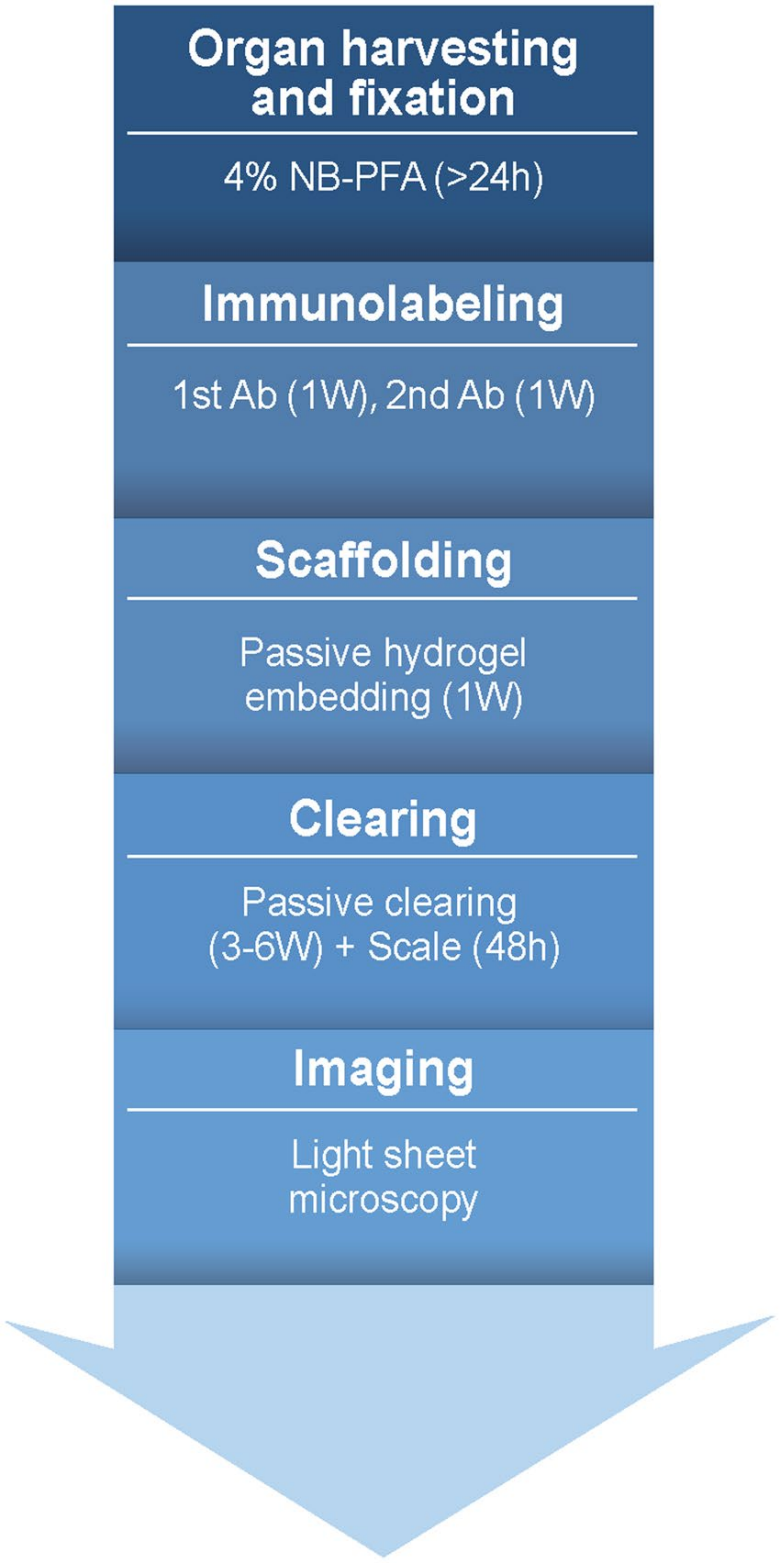
The WOBLI method outline. Fresh tissues were passively fixed in 4% PFA for at least 24 hours. After fixation, tissues were permeabilized and then immunolabeled for lymphatic vessels using LYVE1 antibody. Labeled tissues were scafolded in hydrogel and subsequently cleared and placed in Sca*l*eA2 solution. Light-sheet microscopy was utilized for the three-dimensional imaging of the blood and lymphatic vasculature.

The WOBLI method consists of immunolabeling, hydrogel embedding, tissue clearing and imaging as described below (Fig. 1). This method allows preservation of tissue structure and fluorescence of both reporter gene and the targeted antibody. Clearing was concluded using Sca*l*eA2 solution that replaced the high-cost FocusClear reagent used in the CLARITY method, thus making the whole process more affordable.

#### Step 1: Immunofluorescence labeling

Mice were euthanized with pentobarbital (80 mg/kg i.p.). Transcardial perfusion was not used. Organs were rapidly removed, fixed in 4% NB-PFA at 4 °C for at least 24 hours and placed in permeabilization solution (0.2% Triton X-100 in PBS) for 8 hours. Tissue samples were then blocked with blocking buffer (10% NGS and 0.05% Triton X-100 in PBS) for 20 hours at 4 °C, incubated for 1 week at 4 °C with rabbit anti mouse LYVE1 antibody, washed twice (0.1% NGS and 0.05% Triton X-100 in PBS; 12 hours each), incubated with donkey anti rabbit Alexa fluor-647 antibody for 1 week at 4 °C, and washed again as mentioned above. Primary and secondary antibodies were diluted 1:100 in antibody buffer (5% NGS and 0.05% Triton X-100 in PBS).

#### Step 2: Hydrogel embedding and clearing

Hydrogel embedding was performed according to the CLARITY protocol^19^ as follows. Immunolabeled samples were immersed in hydrogel solution (4% acrylamide, 0.025% bis-acrylamide, 0.25% VA-044 initiator and 4% PFA in PBS) in 50 ml conical tubes for 1 week at 4 °C for passive diffusion. Sample tubes were then degassed using nitrogen for 5–10 min and immediately incubated at 37 °C for 3 hours or until polymerization. Samples were carved out from the gel in a fume hood and immersed in clearing solution (200 mM boric acid, 4% SDS) at 37 °C for passive clearing (4 weeks for ovaries, 6 weeks for lungs, livers and brains). After complete clearance (Supplementary Figure 1), samples were washed twice with PBST (0.1% Triton X-100 in PBS; 24 hours each) and immersed in Sca*l*eA2 solution (4 M urea, 10% glycerol, 0.1% Triton X-100) for 48 h at room temperature.

#### Step 3: Imaging

Samples were imaged using a Zeiss Z1 light-sheet microscope (Zeiss, Germany) equipped with sCMOS PCO edge cameras, detection optics of LD-Plan-Apochromat 20 × 1.0 Corr M32 85 mm VIS-IR (Zeiss-421459-9772-000) nd-1.38, suitable for Sca*l*eA2 method with RI = 1.38. Illumination Optics light-sheet Z.1 10 × 0.2. The aim in this system setup is to match the refractive index of all imaging system components (sample-media-lens), in order to create a high-resolution image. Samples were glued to a dedicated holder and placed in the imaging chamber. Imaging was preformed using dual side illumination (where indicated, projection of data before fusion is presented in Supplementary Figure 2) and tile stitching (where indicated, with overlap of 10%). Vecad tdTomato imaging: excitation, 561 nm, detection filter cube, BP575–615. LYVE1- Alexa fluor-647 imaging: excitation, 638 nm, detection filter cube, LP 660 nm.

#### Step 4: Image processing

Images acquired by the dual side illumination mode, were fused using Zen 2014 sp1 (black edition) software (Zeiss, Germany). Fused images were converted into Arivis Vision 4D format (Germany). Tiles were stitched creating large field of view stacks. Data sets were visualized as projections and 3D volumes.

### Image analysis

Following data reconstruction in Arivis software, the fluorescence intensity data of ovaries was exported as tiff series. The stack was opened in ImageJ, scaled down 10 times in xy dimension (from 0.57 µm/pixel to 5.7 µm/pixel), in order to decrease the memory and computational power needed to analyze the data, and exported as multipage tiff. Machine learning approach that was used for creating a binary mask of the blood vessel compartment was based on user-assisted training in Ilastik software^25^. Filament tracing module of Imaris software (Bitplane, Switzerland) was used for filament (vessels) tracking and extracting numerical data from the binary mask, which was visually evaluated for the match with the original data.

## Results and Discussion

We report here the 3D-visualization of blood and lymphatic vessels networks of ovaries, uteri, livers and lungs using the novel WOBLI method, which combined two existing clearing techniques, CLARITY and Sca*l*eA2. CLARITY provides the artificial polyacrylamide skeleton that is essential for morphology and structure preservation. Sca*l*eA2 (RI = 1.38) on the other hand, offers low-cost and efficient clearing, with fluorescence preservation for at least two years (Supplementary Figure 3), suitable for a large-scale laboratory use. By replacing FocusClear with Sca*l*eA2 we were able to significantly reduce the cost of the method (by approximately 200 fold). Other RI matching solutions such as sRIMS might also replace FocusClear successfully in the CLARITY protocol, however, the effectiveness of sRIMS in this work was not investigated.

The immersion of tissues in clearing solution frequently causes tissue expansion^26,27^. Similarly, in the WOBLI method, tissues expanded in volume after the delipidation process. Based on images of the tissue taken before and after clearing we estimated that the expansion in each dimension is about 15–20%, resulting in ~1.7–2 fold increase in volume (Supplementary Figure 4). Nevertheless, the outlines of the tissue were not altered after clearing by WOBLI, and follicular structures maintained their relative position and spherical shape, consistent with isotropic expansion of the tissue. No further change in volume was observed after immersion in Sca*l*eA2.

We additinally compared signal intensity profiles in three different penetration depths, 100 µm, 0.5 mm and 1 mm (Supplementary Figure 5). Ovaries cleared by WOBLI had comparable fluorescence signal intensity profiles to ovaries cleared by CLARITY, but better profiles compared to ovaries cleared by Sca*l*eA2.

We further showed that the WOBLI method is applicable for a variety of thick tissues (up to 7200 µm × 6400 µm × 2400 µm of a cleared brain specimen, Supplementary Figure 6). In addition, we demonstrated that the antibodies used for immunolabeling successfully penetrated the tissue, fluorescence intensity was preserved and that the clearance allowed imaging throughout the tissue while preserving tissue architecture of blood and lymphatic vessels (Supplementary Figures 6,7). Importantly, the WOBLI method can be applied to fit any tissue of interest with different density and thickness by adjusting parameters such as permeabilization and/or incubation time.

Tissue growth during normal physiology and pathology is accompanied by remodeling of blood vessels to match the changing needs and to maintain the essential supply of oxygen, nutrients and signaling molecules. Along this line, intensive physiological angiogenesis occurs during the female reproductive cycle, and is crucial for proper wound healing and immune response. On the other hand, cardiovascular diseases are often associated with insufficient blood supply leading to ischemia, whereas in cancer, the tumor often stimulates overgrowth of abnormal blood vessels.

The blood vasculature is complimented by the lymphatic system that is essential for maintenance of fluid homeostasis and immune function. It consists of a complex network of lymphatic vessels, lymph nodes and lymphatic organs. Lymphatic vessels are present in most organs, and have a major role in drainage of excess fluid and macromolecules. Additionally, the lymphatic system is involved in pathological conditions including cancer metastasis, inflammation, diabetes and obesity.

**Figure 2:**
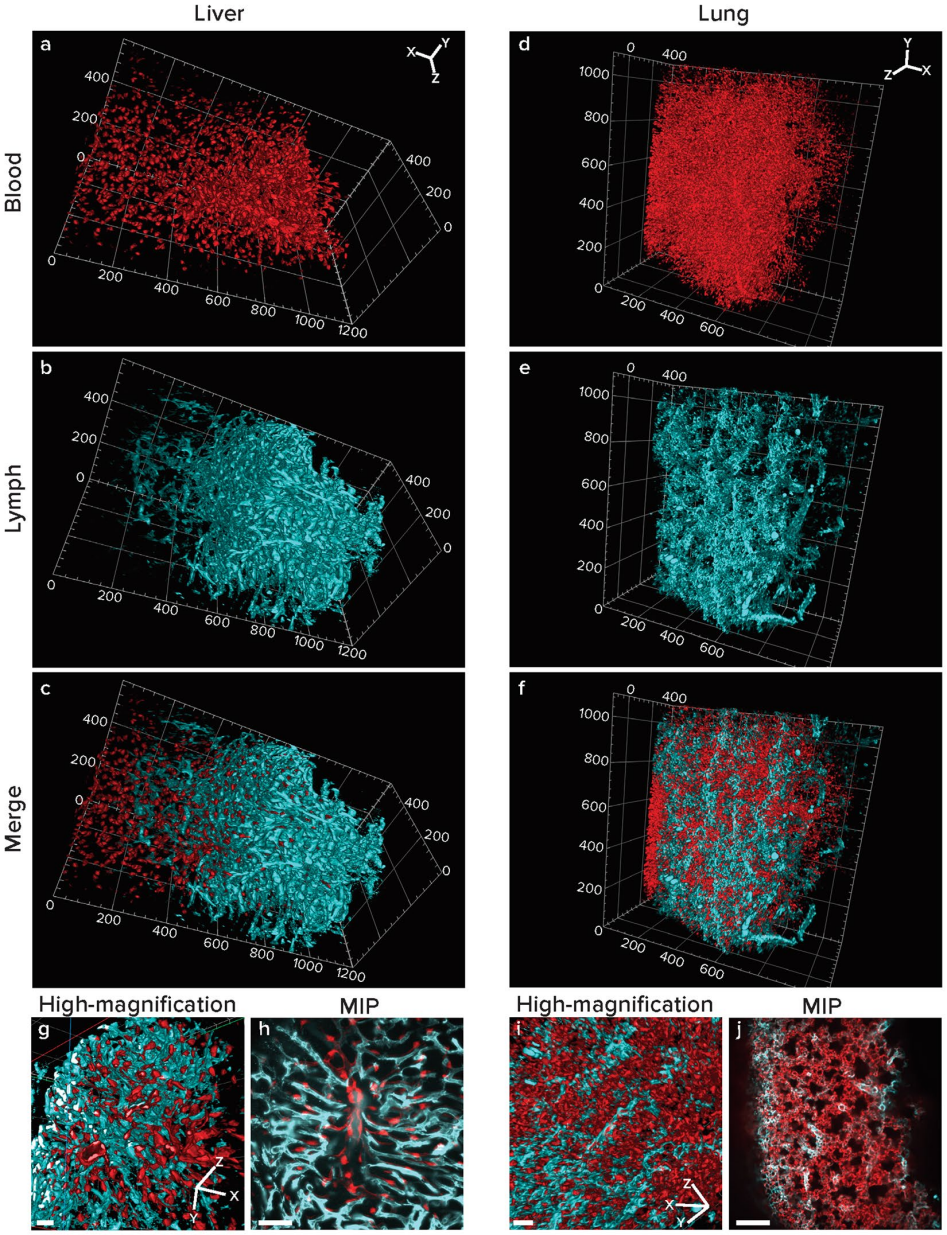
Three-dimensional reconstruction and MIP of slabs for liver and lung. Livers and lungs were harvested from Vecad^cre^/tdTomato^flox/stop/flox^ transgenic mice (blood vessels, red channel) and were labeled with LYVE1 antibody (lymphatic vessels, cyan channel). Organs were cleared and imaged using a light-sheet fluorescent microscope. Imaging was done using dual side illumination and fused (single side illumination is presented in Supplementary Figure 1). Data sets of liver (**a**–**c**) and lung (**d**–**f**) are presented as 3D volumes. Maximal intensity projections (MIP) of slabs (liver: 30 slices/23.1 μm, lung: 3 slices/4.86 µm) show the structure of an hepatic lobule with its mid central vein (**g**) and lung lobules surrounded by vasculature(**j**). High-magnification of rotated images (**g**,**i**) show the continuity of blood and lymphatic vessels. Scale bar: MIPs - liver 50 μm, lung 100 μm; High magnification images – liver and lung 100 μm. Image dimensions: liver 1200 µm × 600 µm × 600 µm, lung 1200 µm × 1000 µm × 400 µm, XYZ (whereas the XY plane is perpendicular to the objective lens). Image dimensions of zoomed in, cropped and clipped images (**c**,**g**): liver 600 µm × 400 µm × 400 µm lung 600 µm × 400 µm × 400 µm, XYZ.

Images of WOBLI cleared livers demonstrated the intimate interaction of blood and lymphatic vessels in this organ (Fig. 2a–c, Supplementary Movie 1). The images obtained provide a sharp visualization of the structure of the hepatic lobules with their mid-central vein (enlarged in Fig. 2h). Such tight interactions between blood and lymphatic vessels were also well demonstrated in the lung (Fig. 2d–f). The continuity of the vessels was visible in the high magnification images (Fig. 2g,j). Imaging the vasculature of these organs in health and disease could contribute to the understanding of different pathologies and further assessment of therapeutic strategies.

**Figure 3:**
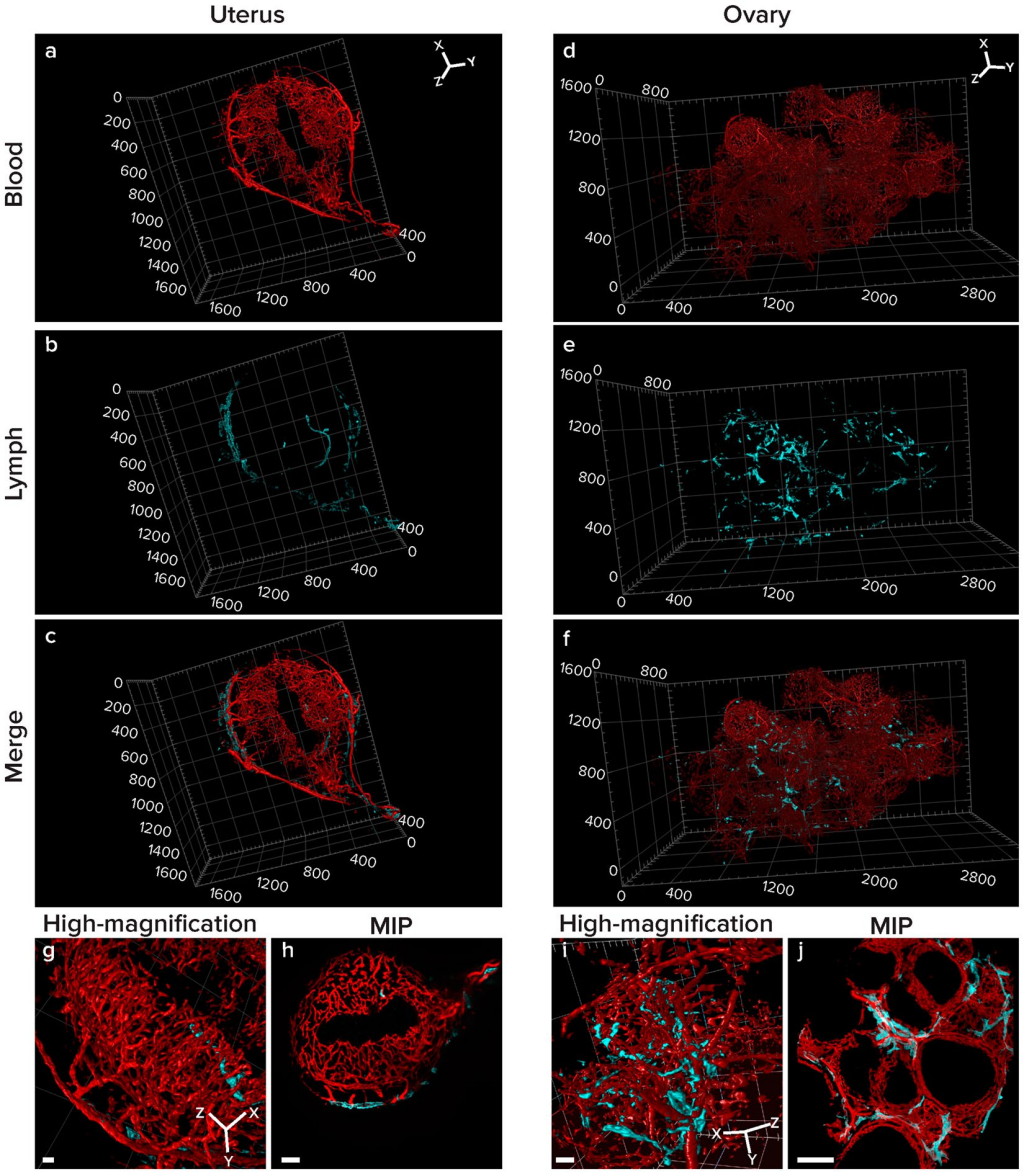
Three-dimensional reconstruction and MIP of slabs of uterus and ovary. Uteri and ovaries were harvested from Vecad^cre^/tdTomato^flox/stop/flox^ transgenic mice (blood vessels, red channel) and were labeled with LYVE1 antibody (lymphatic vessels, cyan channel). Organs were cleared and imaged using a light-sheet fluorescent microscope. Tiles were stitched (10% overlap for stitching) creating large field of view stacks. Data sets of uterus (**a**–**c**) and ovary (**d**–**f**) are presented as 3D volumes. Maximal intensity projections (MIP) of slabs (Uterus: 33 slices/53.46 µm, ovary: 87 slices/140.94 µm) show the uterine spiral arteries, endometrium and myometrium as well as the ovarian follicles surrounded by capillaries. High-magnification of rotated images (**g**,**i**) show the continuity of blood and lymphatic vessels. Scale bar: MIPs - uterus 100 µm, ovary 200 µm; High magnification images – liver and lung 100 μm. Image dimensions: uterus 1200 µm × 1600 µm × 400 µm, ovary 3200 µm × 1600 µm × 1200 µm, XYZ (whereas the XY plane is perpendicular to the objective lens). Image dimensions of zoomed in, cropped and clipped images (**c**,**g**): uterus 900 µm × 800 µm × 200 µm, XYZ ovary 800 µm × 800 µm × 1000 µm.

The vasculature of the uterus undergoes extensive remodeling during the estrous cycle as well as during pregnancy. Our protocol enabled the distinct visualization of the spiral arteries of the uterus, as well as the lymphatic vessels that are associated with them (Fig. 3a–c, Supplementary Movie 2). Immunolabeling with LYVE1 demonstrated that the presence of lymphatic vessels is restricted to the myometrium while the blood vessels are found in both, the myometrium and the endometrium as previously reported^28,29^. Modifications in the endometrial vasculature and the spiral arteries are associated with the reproductive cycle and are essential for nourishing the placenta of the growing fetus during pregnancy. In addition, the lymphatic system plays a significant role in the homeostasis of fluids in embryo implantation and in trafficking of immune cells to the uterus^30^.

Similarly to the uterus, both blood and lymphatic vasculature remodel extensively during development of the ovarian follicle^29,31,32^. Three-dimensional images of cleared ovaries, obtained by the use of our WOBLI protocol (Fig. 3d–f, Supplementary Movie 3), revealed the detailed features of the vasculature in variable stages of follicular development. Potentially, this approach could be used to track structural and functional changes of the blood and lymphatic vessels during ovulation and corpus luteum formation, two processes characterized by intensive angiogenesis.

**Figure 4:**
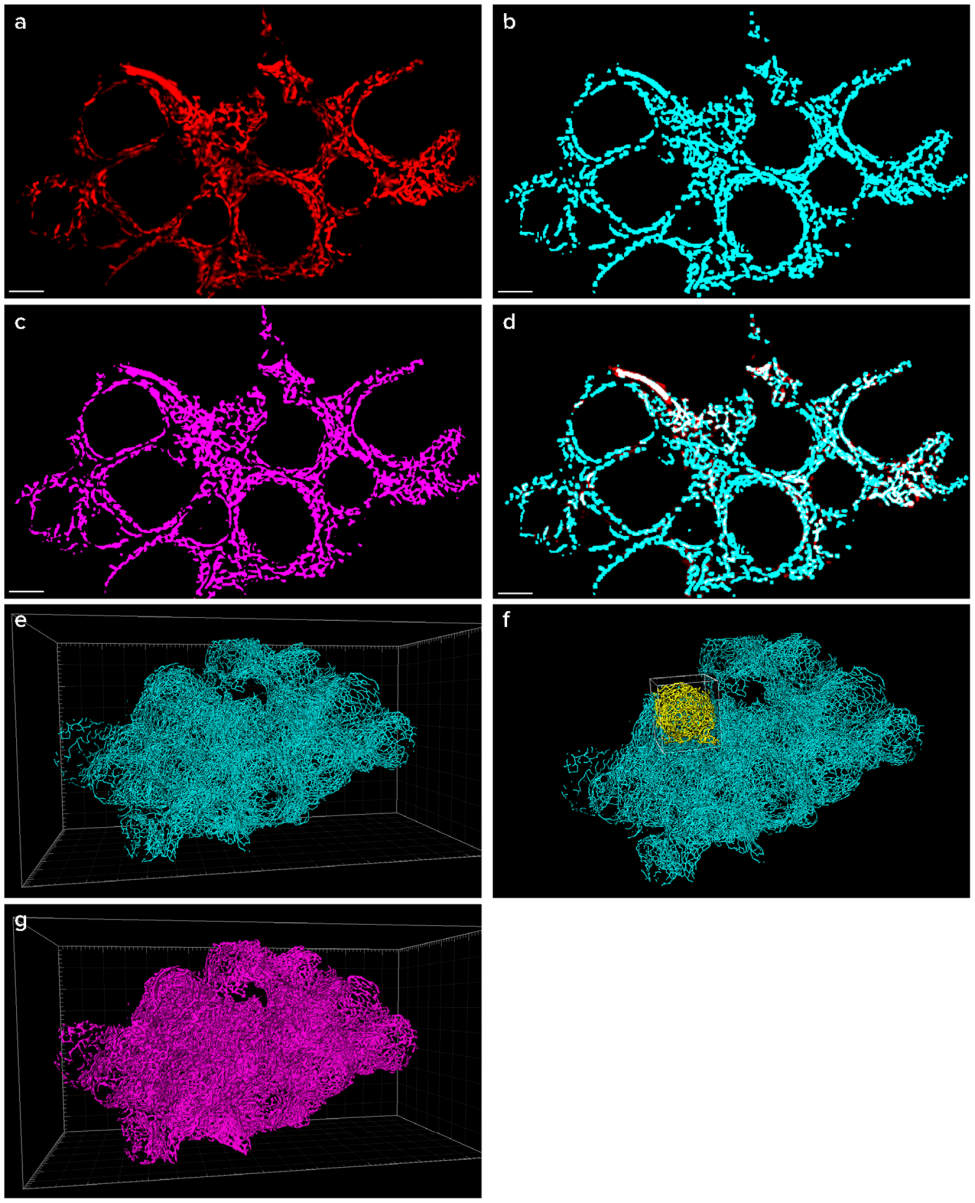
Quantitative analysis of blood vessels in the ovary. A mask was created on the original data of ovarian blood vessels using Ilastik software. From that mask, filaments were calculated using Filament tracing module of Imaris software. A representative slice (**a**), the filament tracing (**b**), the mask created (**c**) and an overlay of the original data with the filaments (**d**) demonstrating high compatibility, are presented (scale bar 300 µm). A 3D reconstruction of the filaments, a mask of the whole ovary (**e**,**g**) and a selected single follicle (**f**) are shown. Numerical parameters calculated from the filament analysis are presented in Table 1.

The extraction of numerical data describing complex morphology of blood vessels networks is a challenging task^33^. Our analysis on the ovarian WOBLI images, described herein, allowed the generation of an accurate binary, 3D mask on the original data. The mask was further used to create a numerical analysis of 3D filament network. (Fig. 4a–d, Supplementary Movie 4). The parameters extracted from the filament analysis included the total vessel length, mean vessel diameter, vessel straightness, and the total number of branching points (Table 1). All the parameters were calculated both for a single follicle and the entire ovary (n = 2). (Fig. 4f, Table 1). These results demonstrate that the combination of the WOBLI with quantification methods could provide a powerful tool for complex network analysis in different tissues, under physiological as well as pathological conditions.

**Table 1:** Numerical parameters calculated from the filament analysis.

	Entire ovary I	Entire ovary II	Single follicle (ovary II)
Total Vessel Length [µm]	1623250	1136400	45390
Vessel Mean Diameter [µm]	14.27 (± 4.32)	13.15 (± 4.17)	11.02 (± 3.32)
Vessel Straightness*	0.89 (± 0.14)	0.86 (± 0.15)	0.85 (± 0.15)
Total Number of Branching Points	39835	19554	776

*The vessels straightness parameter is a measure of the curvature of the vessel and it is calculated by dividing the distance between two branching points by the length of the filament so a straight vessel will have the value of 1.

**Numbers in brackets indicate standard deviation values.

The ability to image blood and lymphatic vessels network as an intact 3D structure, combined with numerical analysis, offers the possibility for an exact and accurate evaluation of their construction and function. Such detailed evaluation could not be achieved by 2D analysis. Specifically, the three-dimensional visualization of both, blood and lymphatic vessels by WOBLI demonstrate the potential of this tool in improving our understanding of these systems. WOBLI allows the assessment of the complexity and connectivity of the vasculature. In conclusion, the WOBLI protocol described herein provides an accessible method to generate transparent organs/thick tissue slices and enables high resolution 3D-imaging of lymphatic and blood vessels. Adaptation of the combined CLARITY and Sca*l*eA2 protocol for systems other than blood and lymphatic vessels, will require the calibration of different sets of specific antibodies and/or endogenous fluorescent markers.

### Declarations

Experiments were carried out in accordance with the Israeli law and the Weizmann Institute approved guidelines. All experimental protocols were reviewed and approved by the Weizmann Institutional Animal Care and Use Committee.

## Electronic supplementary material

supplementary information

Supplementary Movie 1 Liver

Supplementary Movie 2 Uterus

Supplementary Movie 3 Ovary

Supplementary Movie 4 Quantification
