# Supplementary material for: Whole Organ Blood and Lymphatic Vessels Imaging (WOBLI)
Source: Sci Rep. 2018 Jan 23;8:1412. doi: 10.1038/s41598-018-19663-w (PMC5780490; doi:10.1038/s41598-018-19663-w)
Supplement: Supplementary file 1 — supplementary information [file 41598_2018_19663_MOESM1_ESM.pdf]

Whole Organ Blood and Lymphatic Vessels Imaging (WOBLI)

Roni Oren[#,1], Liat Fellus-Alyagor[#,1], Yoseph Addadi[2], Filip Bochner[1], Hila Gutman[3], Shani Blumenreich[4], Hagit Dafni[5], Nava Dekel[1], Michal Neeman[1], Shlomi Lazar[*,3]

[1]Department of Biological Regulation, Weizmann Institute of Science, Rehovot 76100, Israel.

[2] Department of Life Sciences Core Facilities, Weizmann Institute of Science, Rehovot 76100 Israel.

[3] Department of Pharmacology, Israel Institute for Biological Research, Ness Ziona 74100, Israel.

[4]Department of Biomolecular sciences, Weizmann Institute of Science, Rehovot 76100, Israel

[5] Department of Veterinary resources, Weizmann Institute of Science, Rehovot 76100, Israel

# equal contribution

Supplementary methods

WOBLI method is applicable for detection of a cytoplasmic marker in adult rat brains

Brains were collected from adult 9 weeks-old male Sprague Dawley rats after perfusion. Brains were then labeled with the astrocytic marker, polyclonal rabbit anti-Glial Fibrillary Acidic Protein (GFAP, 1:1000 dilution, Dako, Denmark). Goat anti-rabbit-

alexa fluor 488 (1: 250, Molecular Probes, USA) was used as secondary antibody. Next, brains were cleared as described in the materials and methods section. Experiments were carried out in accordance with the Israeli law, and were approved by the Israel Institute for Biological Research (IIBR) Institutional Animal Care and Use Committee.

Ultra-microscope light sheet imaging

Three-dimensional images of cleared brains were acquired using an ultramicroscope II (LaVision BioTec)[1] operated by the ImspectorPro software (LaVision BioTec). The light sheet was generated by a Superk Super-continuum white light laser (emission 460 nm – 800 nm, 1 mW/nm – 3 (NKT photonics), excitation filter 560/40, emission filter 630\75). Microscope was equipped with a single lens configuration - 4X objective - LVBT 4X UM2-BG, with an adjustable refractive index collar set to the RI of 1.38. Samples were glued to the sample holder and placed in an imaging chamber made of 100% quartz (LaVision BioTec) filled with Sca*l*eA2 solution and illuminated from the side by the laser light. Images were acquired by an Andor Neo sCMOS camera (2,560 × 2,160, pixel size 6.5 µm x 6.5 µm, Andor). Z stacks were acquired in 2 µm steps, larger fields of view were imaged by tiling with 10% overlap. Images were stitched with Arivis software.

Tissue clearing by CLARITY

Tissues were cleared according to the protocol specified by Tomer et. al. [2]. Briefly, 4 months old female Vecad^{cre}/tdTomato$^{flox/stop/flox}$ transgenic mice were transcardially perfused with 20 ml of ice-cold PBS, followed by 20 ml HM solution (4% acrylamide, 0.05% bisacrylamide, 4% PFA, 0.25% (wt/vol) VA-044 thermal initiator, in 1% PBS) at an approximate rate of 10ml/min. organs were then placed in HM solution for 1 day at 4°C. Samples were then de gassed using nitrogen for 5-10 min and immediately

incubated at 37°C for 3 hours or until polymerization. After gel formation, residual gel was removed and samples were immersed in clearing solution (200mM boric acid, 4% SDS) at 37°C for passive clearing. After complete clearing, samples were washed twice in PBST at room temperature, and placed in FocusClear (Celexplorer, Taiwan) for refractive index matching.

Detailed materials information is found in the main text.

Tissue clearing by Sca*l*eA2

Ovaries were harvested from 4 months old female Vecad^{cre}/tdTomato$^{flox/stop/flox}$ transgenic mice and fixed with 4% PFA. Samples were then moved to Sca*l*eA2 solution (4M urea, 10% glycerol and 0.1% Triton X-100) for 6 weeks.

Intensity profile analysis

Images of ovaries cleared by WOBLI, CLARITY, CLARITY with passive hydrogel immersion and Sca*l*eA2 were taken with light sheet microscope. The distance from the lens at the X axis was kept constant. Two-dimensional slices from depths 100 µm, 0.5 mm and 1 mm were chosen for intensity profile analysis. A line was drawn using ImageJ software, and the intensity profile along this line was extracted. The line was always placed from the left tissue edge. If the line was bigger than the tissue seen in the image, it was placed so the tissue will be close to the center of the line. The same line was then placed on the background of the image (where no tissue is imaged) and the average of these values was subtracted from the previous intensity values to form a normalized intensity profile. The normalized intensity values are shown as a function of the distance in pixels on the drawn line.

Supplementary movie 1

Livers were harvested from Vecad^{cre}/ tdTomato $^{flox/stop/flox}$ transgenic mice (blood vessels, red channel) and were labeled with LYVE1 antibody (lymphatic vessels, cyan channel). Livers were cleared and imaged using a light-sheet fluorescent microscope. 3D projection of liver is presented in the movie Image dimensions: 1200µm × 600µm × 600µm XYZ.

Supplementary movie 2

Uteri were harvested from Vecad^{cre}/tdTomato$^{flox/stop/flox}$ transgenic mice (blood vessels, red channel) and were labeled with LYVE1 antibody (lymphatic vessels, cyan channel). Uteri were cleared and imaged using a light-sheet fluorescent microscope. 3D projection of uterus is presented in the movie Image dimensions: 1200µm × 1600µm × 400µm, XYZ.

Supplementary movie 3

Ovaries were harvested from Vecad^{cre}/tdTomato$^{flox/stop/flox}$ transgenic mice (blood vessels, red channel) and were labeled with LYVE1 antibody (lymphatic vessels, cyan channel). Ovaries were cleared and imaged using a light-sheet fluorescent microscope. 3D projection of ovary is presented in the movie. Image dimensions: 3200µm × 1600µm × 1200µm, XYZ.

Supplementary movie 4

Demonstration of the high compatibility between the ovarian blood vessels as shown in the raw data and the Imaris calculated filaments (scale bar, 300µm). Raw data in red, filaments in cyan.

Supplementary figure 1

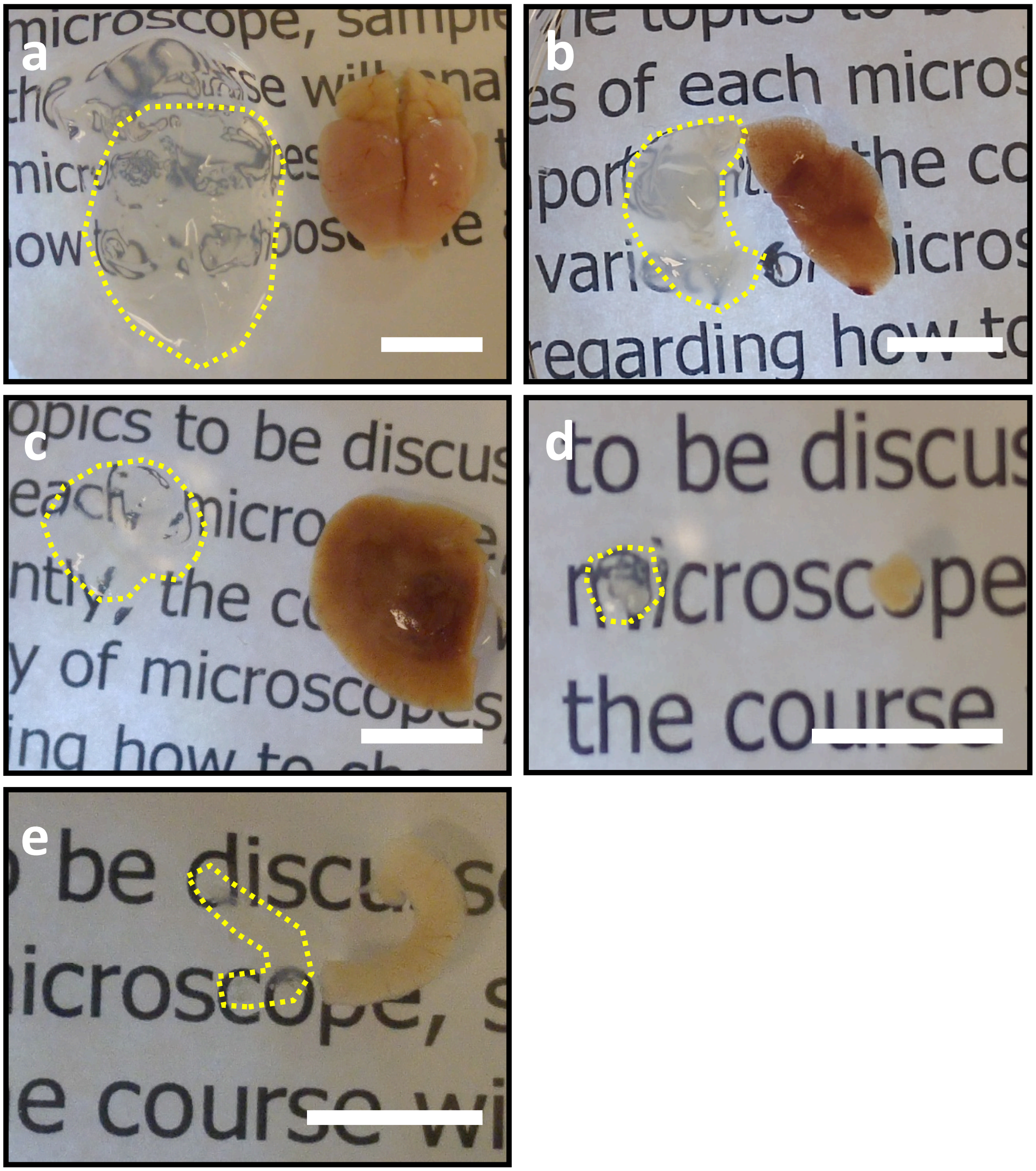

Transparency of intact whole organs after clearing using the WOBLI method.

Images of a brain (a), lung (b), liver (c), ovary (d) and uterus (e) pre and post clearing.

All tissues were labeled and cleared as indicated in the methods section. Dashed yellow line indicated the location of the cleared tissues, (scale bar 5mm).

Supplementary figure 2

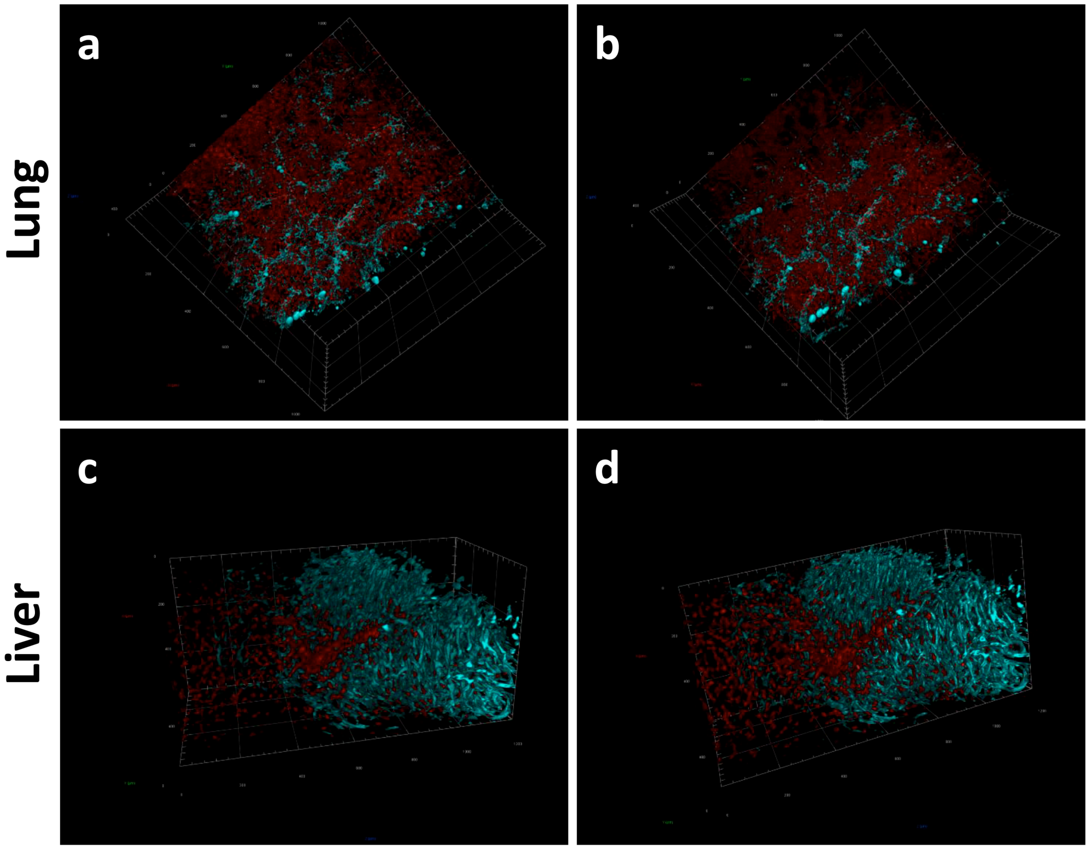

Three-dimensional projections of each of the dual side illuminations Left side illumination (a,c) and right side illumination (b,d) of a cleared lung (a,b) and liver (c,d). Blood vessels are indicated in red, lymphatics are indicated in cyan.

Supplementary figure 3

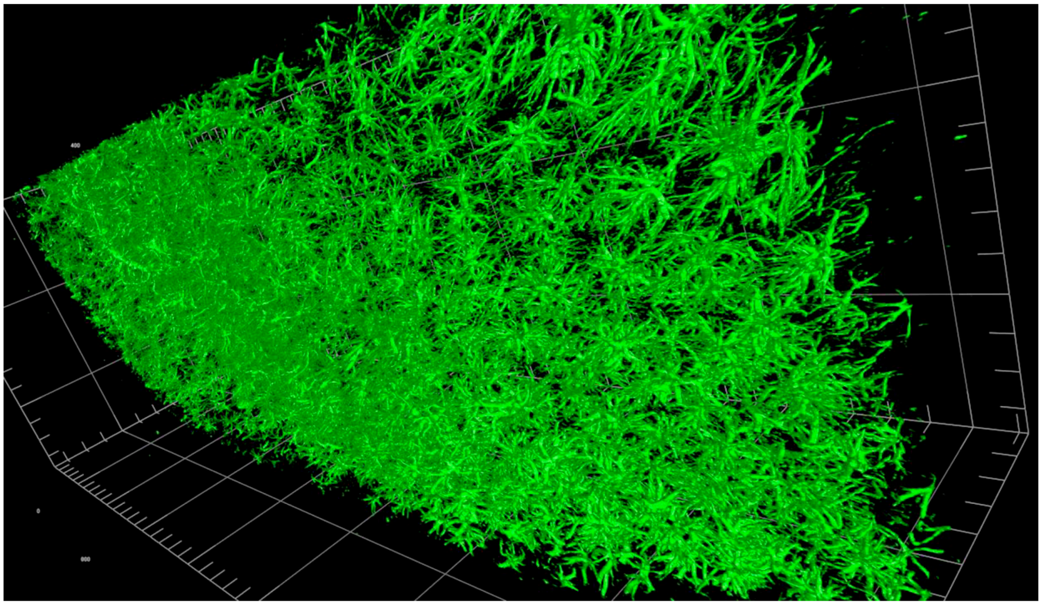

WOBLI method in an adult rat brain with a cytoplasmatic marker. Brains were collected from adult 9 week-old perfused rats and labeled with the astrocytic marker, polyclonal rabbit anti- Glial Fibrillary Acidic Protein. In the next step, brains were cleared as described in the materials and methods section. Imaging was done 2 years after clearing. Image dimensions – 400 μm × 800 μm × 3200 μm (XYZ).

Supplementary figure 4

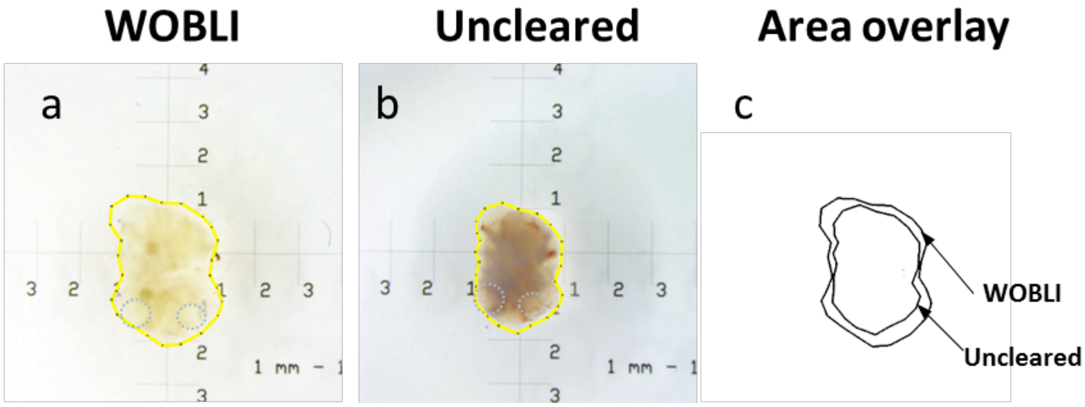

Size comparison of ovaries before and after clearing by WOBLI.

Ovaries after WOBLI clearing process (a) tissue expansion compared to uncleared ovaries (b). Based on calculated tissue area we estimate the change in tissue volume to be 2 fold higher after clearing. The outer borders of the tissue remain similar before and after clearing (c), and the spatial location and shape of the ovarian follicles are preserved (gray circles). These results are consistent with isotropic expansion of tissue.

Supplementary figure 5

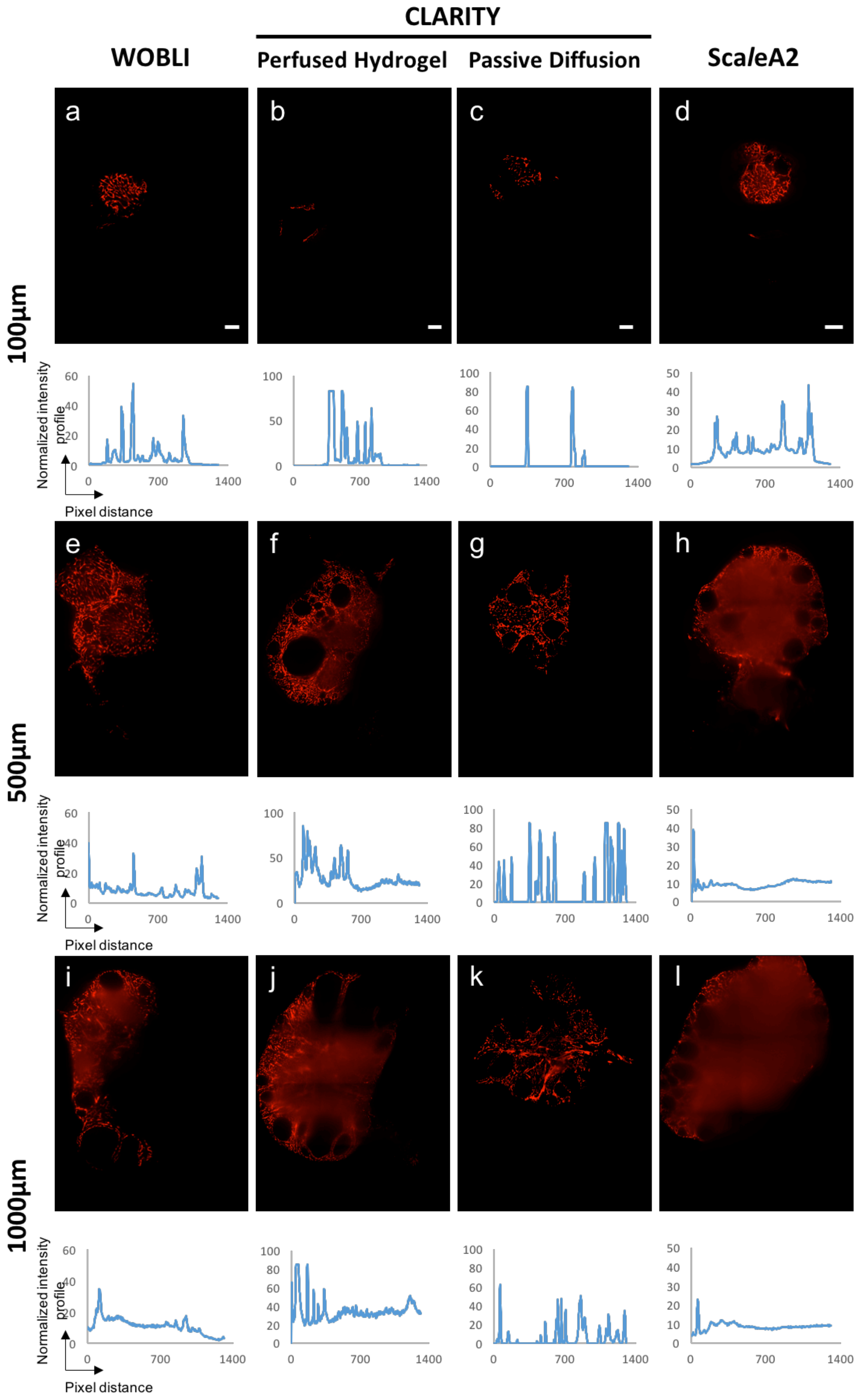

Penetration depth and signal intensity profiles of ovarian vasculature cleared by WOBLI, CLARITY and Sca*l*eA2.

The vasculature of ovaries cleared by WOBLI, CLARITY (either with perfused or passively diffused hydrogel) and Sca*l*eA2 were imaged using a light sheet microscope. Two dimensional sections from 3 different depths in the tissue (100 µm, 0.5mm and 1 mm) are shown for each clearing method (scale bar 200 µm). The apparent differences in the CLARITY protocol images between perfused and passive diffused hydrogel might be attributed to sample variability and handling. For the assessment of signal quality, signal intensity profiles of each 2D section were plotted (as specified in supplementary methods). The intensity profile derived from WOBLI cleared ovaries (a, e, i) was comparable to the CLARITY cleared ovaries (b,c,f,g,j,k) , and showed higher signal relative to Sca*l*eA2 cleared ovaries (d,h,l).

Supplementary figure 6

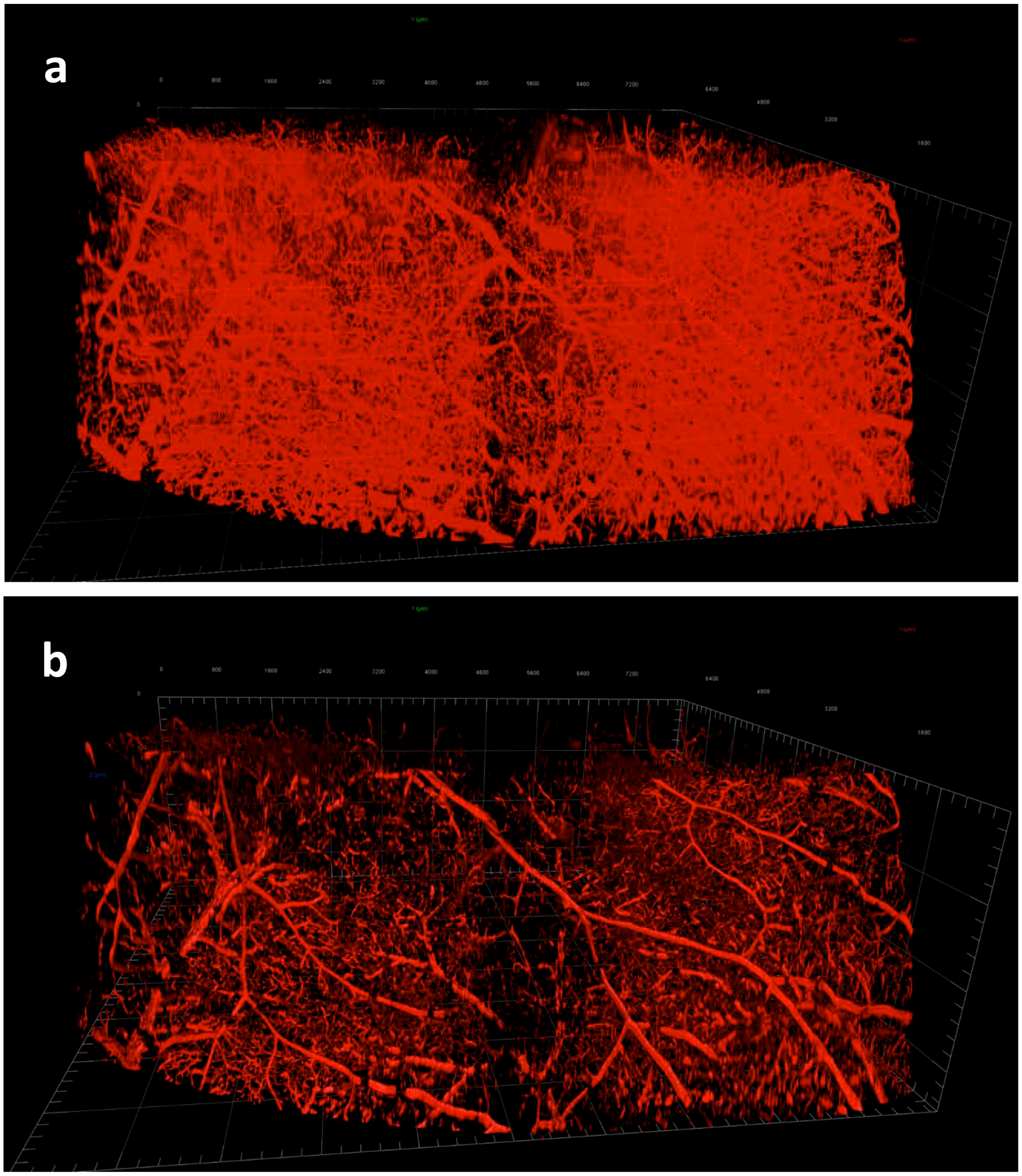

WOBLI application on Vecad^{cre}/tdTomato$^{flox/stop/flox}$ brain. Young, 16 day old Vecad^{cre}/tdTomato$^{flox/stop/flox}$ mice were perfused, and their brains were collected and fixed in 4% PFA. Brains were then cleared as indicated in the methods section. The

background fluorescence (a) reveals the tissue boundaries, the vasculature is indicated in red (b). Image dimensions: 6400 × 7200 × 2400 µm (XYZ).

Supplementary figure 7

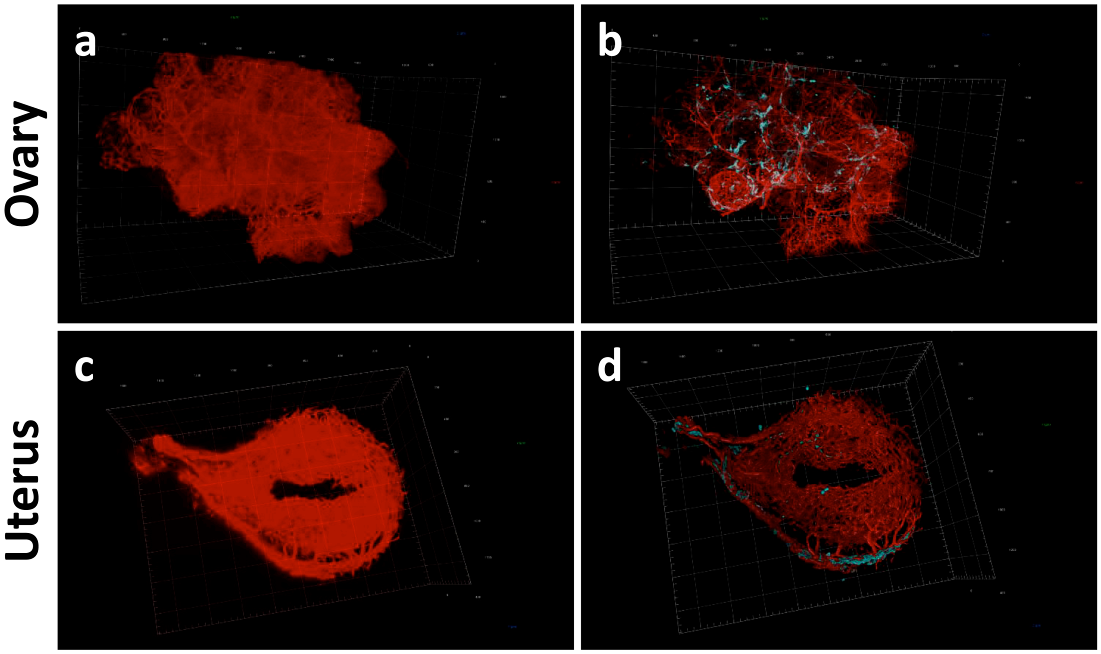

Background fluorescence images indicate tissue boundaries and assist in determining blood vessel distribution in the ovary and uterus. Background fluorescence images of ovary (a) and uterus (c) describes the tissue boundaries and morphology. Three dimensional structure of the lymphatics (cyan) and blood vasculature (red) in the tissue (b,d).

1 Belle, M. *et al.* Tridimensional Visualization and Analysis of Early Human Development. *Cell* **169**, 161-173 e112, doi:10.1016/j.cell.2017.03.008 (2017).
2 Tomer, R., Ye, L., Hsueh, B. & Deisseroth, K. Advanced CLARITY for rapid and high-resolution imaging of intact tissues. *Nat Protoc* **9**, 1682-1697, doi:10.1038/nprot.2014.123 (2014).
